# Mechanisms of Cell Cycle Arrest and Apoptosis in Glioblastoma

**DOI:** 10.3390/biomedicines10030564

**Published:** 2022-02-28

**Authors:** Konstantinos Gousias, Theocharis Theocharous, Matthias Simon

**Affiliations:** 1 Department of Neurosurgery, St. Marien Academic Hospital Lünen, KLW St. Paulus Corporation, 44534 Luenen, Germany; theocharous.theocharis@klinikum-luenen.de; 2 Medical School, Westfälische Wilhelms University of Muenster, 48149 Muenster, Germany; 3 Medical School, University of Nicosia, Nicosia 2414, Cyprus; 4 Department of Neurosurgery, Bethel Clinic, University of Bielefeld Medical School, 33617 Bielefeld, Germany; matthias.simon@evkb.de

**Keywords:** glioblastoma, cell cycle arrest, apoptosis, p53 pathway, Rb pathway, ion channels, nucleocytoplasmic shuttling, Karyopherin a2 (KPNA2), exportin 1 (XPO1)

## Abstract

Cells of glioblastoma, the most frequent primary malignant brain tumor, are characterized by their rapid growth and infiltration of adjacent healthy brain parenchyma, which reflects their aggressive biological behavior. In order to maintain their excessive proliferation and invasion, glioblastomas exploit the innate biological capacities of the patients suffering from this tumor. The pathways involved in cell cycle regulation and apoptosis are the mechanisms most commonly affected. The following work reviews the regulatory pathways of cell growth in general as well as the dysregulated cell cycle and apoptosis relevant mechanisms observed in glioblastomas. We then describe the molecular targeting of the current established adjuvant therapy and present ongoing trials or completed studies on specific promising therapeutic agents that induce cell cycle arrest and apoptosis of glioblastoma cells.

## 1. Introduction

Glioblastoma multiforme (GBM) account for 49% of all primary malignant central nervous system tumors [1] and stand for the most malignant part of the clinical spectrum. The current established therapy consists of a gross total resection when safely feasible, followed by adjuvant radio-, chemo-, or radiochemotherapy and application of tumor treating fields (TTF). Nevertheless, prognosis is still poor, and overall survival after completion of these therapies averages less than 2 years [2]. Each therapeutic modality has severe conceptual limitations. The surgical gold standard for the treatment of tumors in general is a complete tumor resection, which is however not feasible for principal reasons in the case of the infiltrative GBM; the present resection policy is restricted to a gross total tumor removal reflecting a definition of the extension of the neoplasm based on neuroimaging criteria; some aim at so-called supramarginal resections, but microscopic distant satellites after resection may still be left behind after surgery [3,4]. 

Dependent on the molecular background of the patients’ tumor, the efficacy of the chemotherapeutic protocols varies considerably. Furthermore, the chemotherapeutic agents may not accumulate in sufficient levels in the central nervous system due to the blood–brain barrier (BBB) or may have to overcome the resistance acquired by the tumor cells during the therapy. Moreover, we observe a relevant heterogeneity of GBM cells; the adjuvant therapy may be effective for some but not for all mature or stem GBM cells, which may initiate a later tumor recurrence [5]. In addition, drug and radiation toxicity necessitating therapy pauses should not be underestimated. 

Novel therapeutic strategies are urgently needed. To this end, a respectable number of in vitro or in vivo studies focused on targeting the cascades driving excessive proliferation and growth of GBM cells—namely, the pathways of cell cycle and apoptosis. In our work we initially review the machinery of cell cycle and apoptosis in general and then identify the impairments of the relevant pathways specifically observed in GBM. We then discuss some groups of either already established GBM agents or promising novel treatment modalities that may enhance the natural mechanisms of cell cycle arrest and apoptosis of GBM cells. 

## 2. Cell Cycle and Apoptosis

The cell cycle and apoptosis are crucial processes of cell biology, which allow under regular conditions the growth and the homeostasis of the organism. Alterations of these mechanisms may result in pathological conditions, such as tumorigenesis in the case of reduced apoptosis and excessive proliferation. 

We may distinguish between several different cell cycle and metabolic states, such as ongoing cell cycle, quiescence, senescence, apoptosis, or necrosis [6]. Cells under ongoing division undergo the known cell cycle phases, i.e., the G1 gap phase, the S phase (synthesis of DNA), the G2 gap phase, and M phase (mitosis). The phases G1, S, and S2 are alternatively called interphase. During the latter, the cell grows its organelle counts (G1 phase), copies its DNA (S phase), and reorganizes contents in preparation for division (G2 phase). 

Cells in the G0 or resting phase remain in quiescence, which represents a lethargic state characterized by reversible growth arrest and low metabolism [6]. The entrance into the quiescence state allows for resistance to overcome stress and toxic stimuli. After tolerating and repairing the cellular damage, the cells may re-enter a novel cell cycle upon stimulation by specific growth factors, such as cyclin-dependent kinase-2 (CDK2) and E2F [7,8,9,10,11,12,13]. Unlike quiescence, senescence is a state of permanent cell cycle arrest with high cellular metabolism. Both quiescence and senescence are triggered by external and internal signals, such as ionizing radiation, DNA and chromatin damage, endogenic replication stress, cellular stress from reactive oxygen species (ROS), serum starvation, contact inhibition, etc., whereas persistent damage and stress signaling often favor senescence [14,15,16,17]. 

The term apoptosis is used to describe the process of energy-dependent programmed cell death, which leads to the degradation of cell architecture and must not be confused with energy-independent necrosis, which refers to toxic elimination processes after cell death [18,19]. Apoptosis is a physiological mechanism that allows for the necessary removal of severely damaged and dysfunctional cells from the tissues. Discarded cells are replaced through the mitotic activity of (a fraction of) the remaining cells—i.e., tissues need to balance apoptosis and the number of cells not in G0. 

## 3. Machinery of Cell Cycle

**Figure 1:**
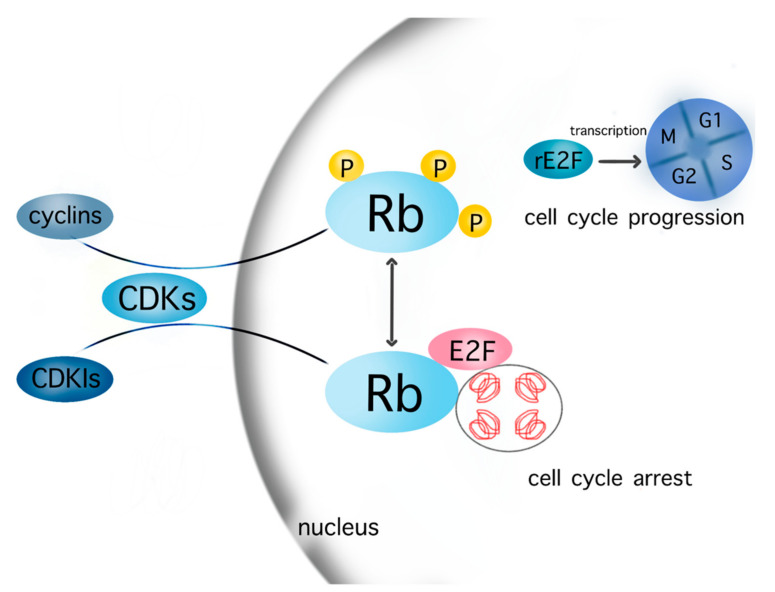
Cell cycle cascades. The cells enter the cell cycle after transcription of released E2F in the nucleus, which is promoted by the phosphorylation of Rb in the context of the interaction between various cyclins–CDKs complexes and Rb. The contrary complex CDKIs–CDKs inhibits the phosphorylation of Rb. Consequently, Rb and E2F are not released from chromatin but still bind to a firm complex, which leads to cell cycle arrest. Abbreviations: CDKs: cyclin-dependent kinases; CDKIs: cyclin-dependent kinases inhibitor; Rb: retinoblastoma, rE2F: released E2F. Most known cyclins: cyclin A, B1, B2, D1, D2, D3, E1, E2, G1; CDKs: CDK1, 2, 4, 6, 9; CDKIs: p16, p21, p27, p57.

The processes of the cell cycle are controlled by two groups of regulatory proteins—i.e., cyclins and cyclin-dependent kinases (CDKs). These proteins act synergistically after their binding, aiming at the phosphorylation of specific members of the pocket protein family, which include p107, p130, and retinoblastoma (Rb) [6,19]. The phosphorylated pocket proteins, such as Rb, are being released from chromatin, thus allowing the transcription of E2F target genes encoding DNA replication signals and the entrance in the S phase [6,19]. The CDKs 2, 4, and 6 as well as the cyclins A2, B1, B2, D1, D2, D3, E1 and E2, and G1 therefore drive the cell through the cell cycle and thereby promote cell growth. CDK/cyclin signaling is antagonized by specific inhibitors of CDKs, (CDKIs) such as p16, p21, p27, and p57. In principle, CDKIs and cyclins compete for binding to CDKs; a complex such as CDK2–cyclin E drives the cell cycle progress; conversely, the CDK4/6–p16 complex induces cell cycle arrest. The relevant cascades of cell cycle are depicted in Figure 1.

## 4. Mechanisms of Apoptosis 

**Figure 2:**
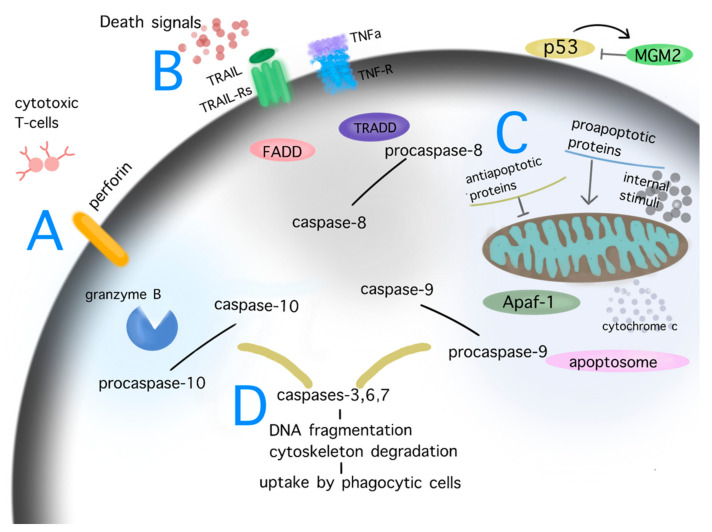
Pathways of apoptosis. Depiction of the cascades of apoptosis. Diverse internal and external stimuli causing persistent damage signaling, such as DNA damage, cellular stress, telomere shortening, ionizing radiation, mitochondrial dysfunction, heat, hypoxia, may trigger the process of apoptosis via the perforin/granzyme (**A**), extrinsic (**B**) or intrinsic/mitochondrial (**C**) pathway. The aforementioned processes activate the final step of apoptosis, named the executive pathway of apoptosis (**D**). During these procedures, initiators (caspases 8, 9, 10) and effectors caspases (caspases 3, 6, 7) are key players in the degradation of critical cell structures until the phagocytosis of the apoptotic cell. Under physiological conditions, p53 activity is controlled by MGM2; in the case of severe permanent damage, signaling p53 is upregulated and triggers the extrinsic and intrinsic pathway of apoptosis. Abbreviations: FADD: Fas-associated protein with death domain; TRADD: tumor necrosis factor receptor type1-associated death domain protein; MGM2: mouse double minute 2; Apaf-1: apoptotic protease-activating factor 1; TNFa: tumor necrosis factor alpha; TNF-R: tumor necrosis factor alpha receptor. Common ligand bindings of the extrinsic pathway are TNFa/TNF-Rs, ApoLs/DRs, TRAIL/TRAIL-Rs, FasL-Fas-R. Known proapoptotic proteins: Bcl-10, Bax, Bak, Bad; antiapoptotic proteins: Bcl-2, Bcl-x, BAG.

The role of physiological apoptosis is to maintain homeostasis of multicellular organisms. Severely damaged cells may induce their own death. Apoptotic cell losses are balanced by the mitotic activity of the remaining cells. The morphological changes during apoptosis comprise the initial cell shrinkage, followed by pyknosis of the cytoplasm and organelles, condensation of chromatin, and degradation of DNA until to the final uptake of the damaged cell by phagocytic cells [18]. The cascades of apoptosis are triggered by either intrinsic or extrinsic signals; external signals may interact with cell surface receptors of TNF and FAS in order to induce the extrinsic or death receptor pathway, whereas intrinsic signaling such as nutrient loss, endogenous stress, heat or cold shock, or telomere shortening may trigger the intrinsic or mitochondrial pathway. An additional pathway of apoptosis may be mediated by perforin/granzyme signaling. All different initial pathways merge to a final executive pathway. The processes of both initial and executive phases of apoptosis are regulated by a group of cysteine proteases, called caspases, which are categorized as initiators (caspases 1, 2, 8, 9, 10) and effectors (caspases 3, 6, 7) according to their phase of activation (initial vs. executive phase of apoptosis). Caspases act proteolytically and cleave proteins at aspartic acid residues. A further regulation of apoptosis during the initial intrinsic mitochondrial pathway is carried out by members of the Bcl-2 family of proteins [20]. The latter family comprises both anti- (Bcl-2, Bcl-x, BAG) and pro- apoptotic (Bcl-10, Bax, Bak) proteins. Figure 2 shows the pathways of apoptosis—namely, the intrinsic, extrinsic, and perforin/granzyme pathway (Figure 2). 

## 5. Regulatory Pathways of Cell Cycle and Apoptosis

### 5.1. Rb Pathway (Retinoblastoma)

The Rb protein is a known tumor suppressor and crucial member of the Rb pathway. CDKs, CDKIs, cyclins, members of the pocket protein Rb family, and the E2F transcription factor play prominent roles in the latter pathway. Following phosphorylation, Rb proteins become inactivated, are released from chromatin, and allow for the transcription of E2F target genes, which in turn, induces the progression of the cell cycle. As mentioned above, CDKIs compete with cyclins for CDK binding; a CDK–cyclin complex induces phosphorylation and thereby inactivation of Rb, and subsequently cell proliferation, whereas the formation of CDK–CDKI complexes promotes cell cycle arrest. Noteworthy, p16 specifically inhibits CDK4 and 6, whereas the remaining CDKIs may inhibit all CDKs.

Conversely, activated (i.e., non-phosphorylated) Rb binds to chromatin and prevents cell cycle progression by inhibiting the E2F target transcription [21]. E2F target genes encode signals essential for DNA replication and nucleotide biosynthesis, thus promoting cell growth [19,22]. Formation of repressor complexes between the Rb family proteins and E2F transcription factor takes place in the nucleus after nucleocytoplasmic shuttling through proteins of nucleocytoplasmic transport, named karyopherines [23,24,25,26]. 

### 5.2. P53 Pathway

The p53 pathway, another well-known tumor suppressor pathway, is triggered by specific extrinsic or intrinsic stress signals, such as hypoxia, heat or cold shock, spindle damage, and in particular, DNA damage. The activation of p53 protein drives the cell primarily into cell cycle arrest. During this phase, cells appear more resistant and may repair their damage in order to re-enter a novel cell cycle or on the contrary proceed to apoptosis in the case of irreversible DNA damage [27]. Various factors modify the DNA in different ways, such as gamma irradiation, oxidative free radicals, or alkylation of bases. In response to the different damaging agents, different repair mechanisms are also activated by the cells; this in turn activates a different network of genes to alter the p53 protein (phosphorylation, acetylation, or methylation) in order to proceed to senescence or apoptosis [28]. Recently, the p53 pathway has also been reported to play a prominent role in the modulation of mitochondrial functions and in glycolysis, as well as in amino acid, nucleotide, lipid, and iron metabolism [29,30]. 

With respect to its main role—i.e., the regulation of cell cycle—p53 may deplete regulators of cell cycle progression, such as cyclins (A, B1, B2, D) or CDKs (CDK1, 2, 4, 6) and may induce the formation of suppressor complexes such as p21Waf/Cip1 (or o21/CDKN1A), resulting in G1/M or G2/M arrest [21,27]. Upon irreversible damage or stress signals, p53 activates the BH3-members of the Bcl-2 protein family (BAX/BAK), which then induce apoptosis. 

Under physiological conditions, p53 levels and activity are regulated by a E3 protein ligase, called mouse double minute 2 (MDM2), which inhibits the p53 transcriptional activity upon binding and subcellular translocation of p53 from the nucleus to the cytoplasm via exportin-1 (CRM1 or XPO1) [31,32]. 

### 5.3. Remaining Cell Cycle Regulators

Apart from the main regulators of cell cycle—namely p53 and Rb— further pathways are also partially involved in cell proliferation. The most important target protein of the P13K/AKT pathway is mTOR, which induces the biosynthesis of various cyclins, such as cyclin D1, and therefore promotes cell growth [33]. Certain MAPK pathways, such as the ERK, p38, and JNK signaling pathways promote both anti- and pro-apoptotic cascades, depending on their trigger signals [34]. The NF-kB pathway includes four transcription factors (NF-kB1, NF-kB2, RelA/p65, and c-rel), which are translocated into the nucleus to bind to specific sequences of DNA prior to the transcription of genes involved in apoptosis. A further trigger for apoptosis is the physiological shortening of a nucleoprotein series at the end of the chromosomes, called telomeres, which acts as a signal for p53 activation. Eucaryotic organisms are capable of adding new nucleotide complexes to the telomeres using their own RNA through activation of the telomerase reverse transcriptase (TERT) pathway.

Nucleocytoplasmic shuttling is a crucial regulator of cell cycle and apoptosis since a considerable number of proteins involved in the p53 and Rb pathways, such as Rb, p53, p21, p27, NF-kB, c-Myc, and E2F1, are translocated through the nuclear membrane with the help of members of a family of nuclear protein-transporters, called karyopherins [26,35,36,37]. The most studied karyopherin a2/importin unit 2 complex (KPNA2) is thought to mediate the nuclear import of macromolecules (mostly proteins and RNAs) by binding to a specific protein sequence called the nuclear localization signal (NLS), which is translocated through the nuclear pores into the nucleus. After entering the nucleus, the NLS-containing macromolecule is dissociated by RanGTP, and KPNA2 recycles back to the cytoplasm. Protein export requires another specific recognition sequence, called the nuclear export signal (NEL). The latter complex is recognized by another karyopherin, the chromosome region maintenance protein 1/exportin 1 (CRM1/XPO1), which mediates the export of specific cargo proteins [38]. An aberrant subcellular translocation of various pro-apoptotic transcription factors or tumor suppressors may affect their function—i.e., deactivate them or even confer a malignant proliferative behavior [39].

## 6. Aberrant Cell Cycle Progression and Apoptosis in GBM

Dysregulation of a variety of cellular pathways, in particular those involved in the regulation of the cell cycle machinery and apoptosis, is observed in several types of cancer. Subsequently, tumor cells may escape apoptosis or senescence and show excessive proliferation and tumor growth. One can argue that apoptosis and senescence protect against cancer. The most common alterations found in GBM affect p53 [40]; 30% of primary and 65% of secondary GBM express mutated p53 [41]. Both missense and splice site mutations are observed [42]; in the case of the former, several hotspots in the DNA-binding domain—namely, R175, R248, R249, R273, R273, R282, and G245—are most frequently mutated, according to the GBM PanCancer Atlas of The Cancer Genome Atlas (TCGA) [43,44,45]. In addition, methylation of the p53 gene promoter was detected in 21% of primary GBM in one study [46]. Apart from loss or mutation of the p53 gene, further mechanisms that result in p53 inactivation in GBM include impairment of p53 protein stability and suppression of p53 gene expression through amplification of p53 inhibitor genes, such as MDM2 and MDM4 [47,48], genetic deletion and methylation of the p53 inducer ARF [49], genomic loss of ATM, CHEK2 [50], mutation of Parkin [51], overexpression of NFIA, and miR-141-3o [52,53], Bcl2 [54,55], and MIF [56]. 

According to the TCGA data, prominent alterations in the Rb pathway include homozygous deletions or mutations of genes coding for members of the pocket protein family, in particular of Rb, and gene amplification of cell cycle promoters such as CDKs (CDK4 and 6) and cyclin D1 [57]. Mutation, deletion, or methylation of Rb is observed more frequently in secondary GBM [58]. 

The P13K/AKT/mTOR pathway is upregulated in GBM cell lines, such as U138 MG [59]. Homozygous deletions or mutations of PTEN, mutations of P13K, as well as amplification of AKT and FOXO genes are documented in the TCGA [57]. PTEN mutations have been associated with poor survival in GBM patients [60]. 

A higher expression of p38 showed a positive correlation with the WHO grade of malignancy in gliomas, implying also an aberrant activity of the MAPK pathway [61]. Stem cell GBM cells showed self-renewing ability upon phosphorylation of JNK; the systemic administration of small-molecule JNK inhibitors blocks this ability [62]. 

**Figure 3:**
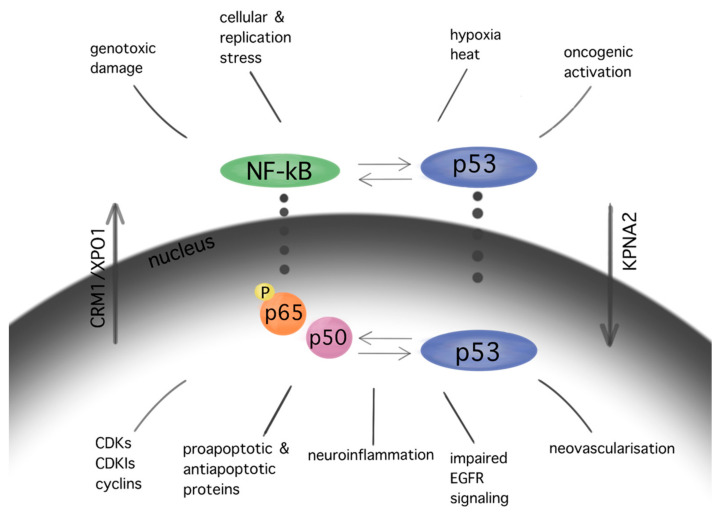
Interactions between pathways of p53 and NF-kB in GBM. Similar stimuli trigger NF-kB and p53 pathways. In turn, several interactions between the aforementioned cascades are observed in multiple levels at their cytoplasmic as well as their nuclear localization, resulting in cell cycle arrest, apoptosis, neuroinflammation, impaired EGFR signaling, and angiogenesis in GBM. The subcellular translocation is performed by karyopherins (nuclear import: KPNA2; nuclear export: CRM1/XPO1).

The NF-kB pathway, which demonstrates anti-apoptotic activity, is upregulated in GBM cells. The NF-kB p65 subunit is overexpressed in gliomas, showing a positive correlation with the WHO malignancy [63]. Inhibition of the NF-kB subunits RelA and c-Rel drives cell cycle arrest and reduction in tumor growth in GBM cells [64]. Significant interactions between NF-kB and p53 cascades in GBM promote cell cycle arrest, apoptosis, neovascularization, impaired EGFR signaling, and neuroinflammation [65] (Figure 3). 

Mutations in the TERT gene promoter underlie a further escape mechanism from apoptosis in GBM; GBM cells maintain the telomere length in the context of increased telomerase activity, and this in turn leads to excessive proliferation. TERT promoter mutations are frequently observed in IDH-wildtype GBM [66,67]. TERT promoter mutations have been correlated with shorter survival [68].

In vitro and in vivo studies showed an aberrant nucleocytoplasmic transport in patients with GBM or GBM cell lines [37,39]. KPNA2 and CRM1 are upregulated in brain tumors, whereas their expression correlated positively with the WHO malignancy grade [38,39]. KPNA2 expression, in particular, showed an inverse correlation with the patients’ overall and progression-free survival. Increased KPNA2 expression in the UM87 GBM cell line was associated with more malignant behavior via activation of the p53 pathway [37]. 

## 7. Principles of GBM Molecular Targeting

In principle, brain carcinogenesis takes advantage of existing cell cycle control pathways, such as p53 and Rb, which after appropriate “cancerous” modification drive uncontrolled tumor growth. Many brain tumor treatments aim at inhibiting the excessive proliferation of tumor cells by triggering stress signaling and thus initiating senescence or the apoptosis cascade. One major trigger is damage of genetic material. In the following sections we discuss the molecular mechanisms targeted by current therapies. Additionally, we report ongoing trials interfering with the cell cycle machinery in brain tumor cells as well as in vitro studies of promising anticancer agents (Table 1).

### 7.1. Current Therapy in GBM and Cell Cycle Control

The current therapy of patients with GBM consists of maximal safe tumor resection followed by adjuvant radiation and chemotherapy. Different types of radiation and chemotherapy are used for newly diagnosed vs. recurrent GBM and for disparate tumor biology. Additional application of alternating electrical (“tumor treating”, TTF) fields appear as a safe and promising additional therapy [69].

Radiation induces a variety of DNA lesions, such as damaged bases and DNA strand breaks. Approximately 1000 single and 40 double strand breaks are produced per Gy per cell [70]. The radiation-induced DNA damage is monitored by the kinases ataxia-telangiectasia mutated (ATM) and ataxia-telangiectasia and Rad3-related protein (ATR), which in turn initiate the DNA damage response, as previously described [71]. As a consequence, the tumor cells initially undergo cell cycle arrest, and in case of a serious unrepairable DNA damage, cell death via mitotic catastrophe and apoptosis [70]. The effect of radiation therapy depends on the total dose, the number of fractions applied [72], as well as the quality of radiation [73]. The initiation of the mitotic catastrophe occurs not immediately after radiation but rather after accumulation of sufficient genetic damage, reflecting the delayed clinical and imaging response of GBM to radiation. 

Combined treatment with temozolomide and TTF prolonged the overall and progression-free survival of patients with newly diagnosed GBM (EF-14 trial) [69]. TTF force dipole alignment and dielectrophoresis of proteins involved in spindle formation and mitosis, such as septin 2, 6, and 7, a/b tubulin, and microtubules of spindles [74]. The impaired formation of microtubules induces cytoplasmic blebbing, mitotic failure, and abnormal chromosome segregation, with subsequent disruption of mitosis and cell death via apoptosis [75].

The cytotoxicity of the alkylating agent temozolomide is mediated among others by the addition of methyl groups at O^6^ sites on guanines in genomic DNA, which in turn causes base mispairing [76]. In more detail, their toxic product O^6^-methylguanine is then paired with thymine instead of cytosine during DNA replication. The mismatched O^6^-methylguanine to thymine base pair is sensed by DNA repair pathways involving the repair proteins MLH1, MSH2, MSH6, and PMS2, which place the tumor cells initially into cell cycle arrest and eventually cause cell death. However, approximately 60% of patients with GBM show resistance to temozolomide, since a nuclear enzyme, named O^6^-methylguanine-DNA methyltransferase (MGMT), removes alkyl groups from the O^6^-position of O^6^-methylguanine and returns the cell into the regular cell cycle mode. The methylation status of the *MGMT* promoter, which silences MGMT expression, has been identified as being a beneficial prognostic predictor in patients undergoing TMZ chemotherapy [77].

Nitrosoureas are anticancer agents used in the therapy of recurrent GBM but also for newly diagnosed GBM with MGMT promoter hypermethylation [78,79]. Lomustine or CCNU, a well-known nitrosourea, transfers its chloroethyl group to the O^6^ sites of guanine on DNA. This causes interstrand and intrastrand cross-linking of DNA, which inactivates DNA synthesis and leads to cell death. Similar to temozolomide, O^6^-methylguanine DNA methyltransferase (MGMT) also reverts the product of CCNU—namely, the of O^6^-chloroethylguanine, removing its alkyl group, restricting the meaningful use of CCNU in patients with methylated MGMT [80].

**Table 1:** Established and experimental therapeutics targeting cell cycle and apoptosis machinery in GBM.

Treatment	Molecular Target	Mechanism of Action	References
Radiation	**Established therapy**	Apoptosis due to RT-induced double-strand breaks of DNA	[69,70,71,72]
	DNA damage response (DDR), p53		
Alkylating agents (TMZ ^1^, Nitrosoureas)	O ^6^ sites on guanines in genomic DNA	Cell death due to base mispairing and DNA repair pathways	[75,76,77,78,79]
TTF ^2^	Septins and microtubules	Apoptosis due to abnormal chromosome segregation	[68,73,74]
	** Experimental therapy **		
Restoration of p53			
Nutlin-3, RG7388	MDM2 ^3^	Upregulation of apoptosis and senescence due to MDM2 blocking	[81] [NCT03158389 ^4^]
Piperidinones	MDM2-p53	Upregulation of apoptosis and senescence due to MDM2 blocking	[NCT03107780 ^4^]
RITA	P53	Cell cycle arrest via restoration of p53 expression	[82]
Restoration of Rb			
Ribociclib	CDK4/6	Cell cycle arrest via restoration of Rb pathway	[83,84,85] [NCT02345824 ^4^]
TG02	CDK9	Cell cycle arrest via restoration of RB path-way	[86] [NCT02942264 and
			NCT03224104 ^4^]
Nat. compounds			
Curcumin	CDKN2A/p16	G1/S arrest via CDKN2a/p16 upregulation and inhibition of Rb phosphorylation	[87]
	Apoptotic proteins	Apoptosis due to increased BAX/BCL2 ratio	[88]
	Cell cycle regulating pathways	Cell cycle arrest due to modulation of JAK/STAT, MAPK, p13k/Akt, Nf-kB	[21]
Moschamine	Intrinsic pathway of apoptosis	Depolarization of mitochondrial membrane	[89]
Flavonoids	CDK	Cell cycle arrest and apoptosis	[90,91]
		in p53- and Rb-dependent manner	
BET ^5^ inhibitors			
JQ1, UM-002	p13k/Akt	P13k/Akt-mediated apoptosis	[92,93,94]
MicroRNAs	microRNA-21	G0/G1 arrest, apoptosis, inhibits chemoresistance to doxorubicin	[95,96]
Benzimidazoles			
Thiabenzole	MCMP2	G2/M arrest via MCMP2 downregulation	[97]
Flubendazole	Intrinsic pathway of apoptosis, Rb, p53, CDKIs	Increase in proapoptotic proteins, p53, CDKIs, downregulation cyclin B1	[98,99]
5-ALA ^6^	Protoporphyrin IX	Apoptosis via increase in BAX/BXL2 and p53 expression	[100,101]
Ion channels inhibitors	Transmembrane proteins	G1 and G2 arrest, upregulation of p27, Bim, p21, downregulation	[102,103]
		of BCL2 and cyclins	
Karyopherin	Nucleocytoplasmic		
Inhibitors	Shuttling		
siRNA	KPNA2 ^7^	P53-dependent cell cycle arrest via siRNA inhibition of KPNA2	[37]
Selinexor	XPO1 ^8^	Subcellular translocation of cell cycle regulators	[104,105] [NCT04421378 ^4^]

^1^ TMZ: temozolomide, ^2^ TTF: tumor treating fields, ^3^ MDM2: mouse double minute 2, ^4^ https://clinicaltrials.gov/ct2/show/, accessed on 30 December 2021, ^5^ BET: bromodomain and extraterminal family proteins, ^6^ 5-ALA: 5-aminolevulinic acid, ^7^ KPNA2: karyopherin-a2 or importin a2, ^8^ XPO1 or CRM1: exportin 1.

### 7.2. Targeting the Cell Cycle Machinery in GBM

Since GBM cells show uncontrolled cell cycle progression due to alterations of the p53 and Rb pathway, many studies have focused on restoring these functions [106]. Mutations of p53 and Rb are the most common sources of impairments, but direct targeting of p53 and Rb mutations is challenging. However, alternative ways of pathways’ modulations, such as inhibition of natural p53 and Rb deactivators, such as MDM2 or CDKIs, may be both feasible and promising [81,107,108,109,110,111]. Nutlin-3 is a MDM2 inhibitor that targets the MDM2–p53 interaction, inhibiting GBM cell growth via upregulation of apoptosis and senescence [109]. A second generation nutlin analogue called RG7388 is currently under evaluation in conjunction with radiation in the context of the NOA-20 trial (NCT03158389, https://clinicaltrials.gov/ct2/show/NCT03158389, accessed on 30 December 2021). Piperidinones, such as AMG232, are further MDM2–p53 interaction inhibitors, which are tested in a phase I clinical trial in primary and recurrent GBM (NCT03107780) (https://clinicaltrials.gov/ct2/show/NCT03107780, accessed on 30 December 2021). Alternative ways of restoring p53 functions are direct blocking of MDM2 expression via siRNA [82] or restoration of p53 expression via a p53 activator, such as RITA [83]. Similarly, targeting the Rb pathway and CDKs or cyclins drives GBM cells to cell cycle arrest in GBM models [84,85]. CDK4 and CDK6 inhibitors, which showed promising activity in various type of cancers [86], are currently under evaluation in the NCT02345824 ongoing GBM trial (https://clinicaltrials.gov/ct2/show/NCT02345824, accessed on 30 December 2021). TG02, a novel CDK9 inhibitor, is being studied in the clinical trials NCT02942264 and NCT03224104 for recurrent and newly diagnosed GBM, respectively (https://clinicaltrials.gov/ct2/show/NCT02942264; https://clinicaltrials.gov/ct2/show/NCT03224104, accessed on 30 December 2021) [87]. 

A variety of natural substances have been identified as being physiological regulators of the cell cycle via p53 and Rb [32]. Compared with synthetic anticancer agents, they demonstrate a diminished drug toxicity and higher permeability through the BBB [32]. The family of natural compounds comprise among others plant derivatives, curcuminoids, coumarins, alkaloids, carotenoids, flavonoids, marine peptides, and natural steroids [19,21]. The biochemical structures of the studied natural compounds but also the remaining therapeutic agents mentioned in this review are shown in the Table 2. Curcumin upregulates CDKN2A/p16 in DBTRG glial cells, which in turn inhibits phosphorylation of Rb, which leads to a G1/S cell cycle arrest [88]. An increased BAX/BCL2 ratio is also caused by curcumin, inducing apoptosis in a p53-dependent manner via intrinsic mitochondrial pathways [90]. In addition, curcumin is reported to modulate the JAK/STAT, MAPK, p13k/Akt, and NF-kB pathways in favor of cell cycle arrest [21]. Flavonoids such as alkylaminophenol [91] and tectorigenin [89] are metabolites of plants, which promote a p53-, Rb-, and CDK-mediated cell cycle arrest and apoptosis [89,91]. An additional plant compound, named moschamine, activates the intrinsic pathway of apoptosis via dysregulation of the mitochondrial membrane potential, whereas the combined exposure of GBM cell lines to moschamine and temozolomide promotes a stronger cell cycle arrest compared with sole temozolomide exposure [92].

The bromodomain and extraterminal (BET) family proteins are epigenetic regulators of gene transcription by binding via their two tandem bromodomains to lysine-acetylated histones. Since BET proteins regulate the transcription of specific oncogenes as well as cell cycle related genes [93,94,112] they have been investigated as potential therapeutic targets in various cancers [113]. BET inhibitors such as JQ1 [95] induce apoptosis in glioma stem cells by modulating P13K/AKT. A novel BET inhibitor, UM-002, reduced the cell proliferation in patient-derived xenograft GBM cell lines GBM22 and GBM39 [112]. MicroRNAs are non-coding RNAs that regulate gene expression of cell cycle regulatory pathways [96]. Downregulation of microRNA-21 induces in GBM cell lines a G0/G1 cell cycle arrest and increased apoptosis and inhibits chemotherapeutic resistance to doxorubicin [114].

Reassigning a novel role to already established drugs known to be safe is a potentially promising concept in medical oncology. The benzimidazole carbamate family compounds were initially used for the treatment of anthelminthics, but they have shown additional anticancer behavior [97,115]. Hu et al. analyzed the effect of thiabenzole on GBM cell lines (P3, U251, LN229, A172, and U118MG). Thiabenzole was found to induce a G2/M arrest in GBM cell lines via downregulation of mini-chromosome maintenance protein 2 [98]. Flubendazole induces apoptosis via increasing the expression of proapoptotic proteins; in addition, cell cycle arrest is being promoted through downregulation of cyclin B1 and upregulation of p53 and CDKIs, such as p21 in GBM cells [99,116]. Recently, antipsychotic drugs have emerged as potential anticancer agents, whereas 12 candidate substances have been identified [117,118]. Treatment with haloperidol, in particular, has been reported to promote G2/M cell cycle arrest in the U87 GBM cell line [119].

5-Aminolevulinic acid (5-ALA), which induces accumulation of the protoporphyrin IX in GBM cells, is well known as the main diagnostic agent that differentiates the tumor-infiltrated tissue from adjacent healthy brain parenchyma during fluorescence-guided brain surgery [100]. Recently 5-ALA has been assigned a new role either in the context of photodynamic therapy [101] or as a direct cytotoxic agent for GBM [120]. Jalili-Nik et al. report a reduction in Bcl-2 and an increase in Bax and p53 expression, and therefore an increase in apoptotic cells, in the U87MG GBM cell line after in vitro application of 5-ALA [120]. 

Trans- and intracellular shuttling is a fundamental process enabling crucial cell functions, such as the regulation of cell cycle [37,121]. Transmembrane ion channels regulate the responses of the cells to external stimuli, whereas karyopherins translocate macromolecules through the nuclear envelope. Various ion channels, such as Kv10.1, NaV1.6, VDAC2, or CLIC1 are dysregulated in GBM [102,103,114]; higher expressions of TRM3, P2RX4, or CLIC1 are linked to poorer survival [103,114,122]. Since dysregulated ion channels drive tumorigenesis and cell proliferation of GBM cells, their inhibition leads to senescence or apoptosis. Inhibition of the ether-a-go-go-related gene encodes the pore-subunit of K^+^ channel Kv11.1 via siRNA-mediated apoptosis in GBM cell lines [123]. In vitro suppression of the Ca^2+^-activated K^+^ channel BK via its inhibitor, called iberiotoxin, induced S phase arrest and apoptosis [104].

**Figure 4:**
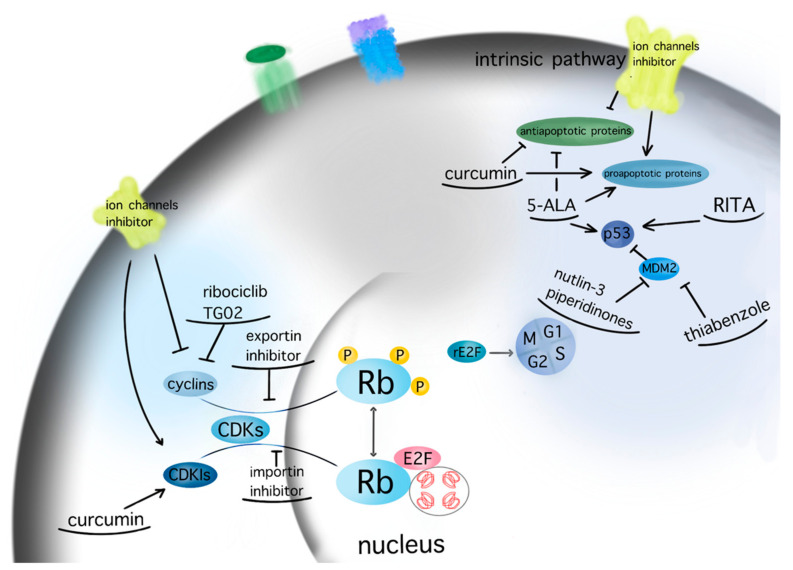
Molecular targeting of GBM therapeutic agents. The current figure depicts the molecular targets of potential therapeutic agents for GBM within the pathways of apoptosis and cell cycle (arrow: upregulation; line: downregulation).

Karyopherins are essential in cell cycle control, as they translocate relevant transcription factors, such as E2F1 and tumor suppressors, as well as oncogenes, through the nuclear envelope [26]. SiRNA-mediated silencing of the most well-characterized importin, karyopherin a2, in U87MG GBM cell line was found to induce cell cycle arrest and apoptosis in a p53-dependent manner [37]. Inhibition of the importin XPO1 or CRM1 via selinexor has reduced proliferation and prolonged survival in GBM animal models [105]. A phase 2 study on efficacy, safety, and intratumoral pharmacokinetics of selinexor monotherapy in recurrent GBM (KING Trial) [124] concluded that there was a clinically relevant response in patients with GBM to a 80 mg weekly dose of selinexor in terms of prolonged progression-free survival [124]. The follow-up study NCT04421378 analyses the effect of selinexor in combination with standard of care therapy for newly diagnosed or recurrent GBM (https://clinicaltrials.gov/ct2/show/NCT04421378, accessed on 30 December 2021). A graphic presentation of the target points of the aforementioned therapeutic agents is given in Figure 4.

## 8. Conclusions

GBM formation involves disruption of proper cell cycle control and escape from programmed cell death—i.e., apoptosis. Mutations in crucial genes such as p53 and Rb, epigenetic alterations, and dysregulation of signaling pathways preclude quiescence and senescence as well as apoptosis. Established chemotherapies for GBM and radiation interfere with tumor growth by affecting cell cycle control and promoting apoptosis. Hence, therapeutic strategies targeting the underlying molecular machinery may have considerable potential. As a testament to the complexity of these regulatory mechanisms, many different concepts and compounds are currently under investigation. Some agents are tested in cell culture and animal models, while other compounds are already being evaluated in clinical trials. No clear favorite strategy or agent however has emerged so far. 

## Figures and Tables

**Table 2:** Biochemical structures of potential therapeutic agents in GBM.

Therapeutic Agents	Biochemical Structure
Nutlin-3	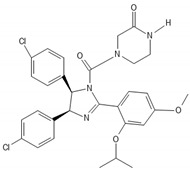
RITA	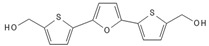
Ribociclib	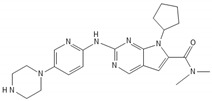
TG02	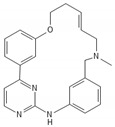
Curcumin	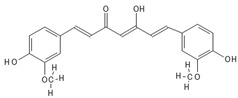
Moschamine	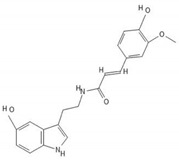
Flavonoids	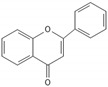
JQ1	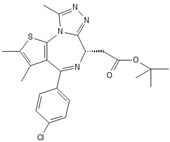
UM-002	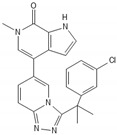
Thiabenzole	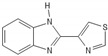
Flubendazole	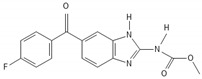
5-ALA	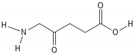
Haloperidol	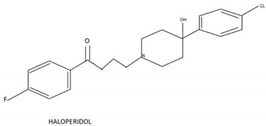
Selinexor	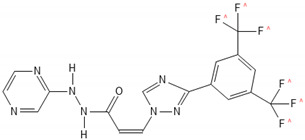

## References

[1] Ostrom Q.T., Cioffi G., Waite K., Kruchko C., Barnholtz-Sloan J.S. (2021). CBTRUS Statistical Report: Primary Brain and Other Central Nervous System Tumors Diagnosed in the United States in 2014-2018. Neuro-Oncology.

[2] Poon M.T.C., Sudlow C.L.M., Figueroa J.D., Brennan P.M. (2020). Longer-term (>/= 2 years) survival in patients with glioblastoma in population-based studies pre- and post-2005: A systematic review and meta-analysis. Sci. Rep..

[3] Vivas-Buitrago T., Domingo R.A., Tripathi S., De Biase G., Brown D., Akinduro O.O., Ramos-Fresnedo A., Sabsevitz D.S., Bendok B.R., Sherman W. (2021). Influence of supramarginal resection on survival outcomes after gross-total resection of IDH-wild-type glioblastoma. J. Neurosurg..

[4] Wang L.M., Banu M.A., Canoll P., Bruce J.N. (2021). Rationale and Clinical Implications of Fluorescein-Guided Supramarginal Resection in Newly Diagnosed High-Grade Glioma. Front. Oncol..

[5] Glas M., Rath B.H., Simon M., Reinartz R., Schramme A., Trageser D., Eisenreich R., Leinhaas A., Keller M., Schildhaus H.U. (2010). Residual tumor cells are unique cellular targets in glioblastoma. Ann. Neurol..

[6] Pack L.R., Daigh L.H., Meyer T. (2019). Putting the brakes on the cell cycle: Mechanisms of cellular growth arrest. Curr. Opin. Cell Biol..

[7] Yao K., Wang Q., Jia J., Zhao H. (2017). A competing endogenous RNA network identifies novel mRNA, miRNA and lncRNA markers for the prognosis of diabetic pancreatic cancer. Tumour Biol..

[8] Kwon J.S., Everetts N.J., Wang X., Wang W., Della Croce K., Xing J., Yao G. (2017). Controlling Depth of Cellular Quiescence by an Rb-E2F Network Switch. Cell Rep..

[9] Hahn A.T., Jones J.T., Meyer T. (2009). Quantitative analysis of cell cycle phase durations and PC12 differentiation using fluorescent biosensors. Cell Cycle.

[10] Spencer S.L., Cappell S.D., Tsai F.C., Overton K.W., Wang C.L., Meyer T. (2013). The proliferation-quiescence decision is controlled by a bifurcation in CDK2 activity at mitotic exit. Cell.

[11] Dong P., Zhang C., Parker B.T., You L., Mathey-Prevot B. (2018). Cyclin D/CDK4/6 activity controls G1 length in mammalian cells. PLoS ONE.

[12] Sakaue-Sawano A., Kurokawa H., Morimura T., Hanyu A., Hama H., Osawa H., Kashiwagi S., Fukami K., Miyata T., Miyoshi H. (2008). Visualizing spatiotemporal dynamics of multicellular cell-cycle progression. Cell.

[13] Cappell S.D., Chung M., Jaimovich A., Spencer S.L., Meyer T. (2016). Irreversible APC(Cdh1) Inactivation Underlies the Point of No Return for Cell-Cycle Entry. Cell.

[14] Daigh L.H., Liu C., Chung M., Cimprich K.A., Meyer T. (2018). Stochastic Endogenous Replication Stress Causes ATR-Triggered Fluctuations in CDK2 Activity that Dynamically Adjust Global DNA Synthesis Rates. Cell Syst..

[15] Arora M., Moser J., Phadke H., Basha A.A., Spencer S.L. (2017). Endogenous Replication Stress in Mother Cells Leads to Quiescence of Daughter Cells. Cell Rep..

[16] Barr A.R., Cooper S., Heldt F.S., Butera F., Stoy H., Mansfeld J., Novak B., Bakal C. (2017). DNA damage during S-phase mediates the proliferation-quiescence decision in the subsequent G1 via p21 expression. Nat. Commun..

[17] Yang H.W., Chung M., Kudo T., Meyer T. (2017). Competing memories of mitogen and p53 signalling control cell-cycle entry. Nature.

[18] Elmore S. (2007). Apoptosis: A review of programmed cell death. Toxicol. Pathol..

[19] Fan H.C., Chi C.S., Chang Y.K., Tung M.C., Lin S.Z., Harn H.J. (2018). The Molecular Mechanisms of Plant-Derived Compounds Targeting Brain Cancer. Int. J. Mol. Sci..

[20] Adams J.M., Cory S. (2018). The BCL-2 arbiters of apoptosis and their growing role as cancer targets. Cell Death Differ..

[21] Wong S.C., Kamarudin M.N.A., Naidu R. (2021). Anticancer Mechanism of Curcumin on Human Glioblastoma. Nutrients.

[22] Knudsen E.S., Wang J.Y. (2010). Targeting the RB-pathway in cancer therapy. Clin. Cancer Res..

[23] Lazzerini Denchi E., Attwooll C., Pasini D., Helin K. (2005). Deregulated E2F activity induces hyperplasia and senescence-like features in the mouse pituitary gland. Mol. Cell. Biol..

[24] Danaei G., Vander Hoorn S., Lopez A.D., Murray C.J., Ezzati M., Comparative Risk Assessment collaborating g. (2005). Causes of cancer in the world: Comparative risk assessment of nine behavioural and environmental risk factors. Lancet.

[25] Jiao W., Datta J., Lin H.M., Dundr M., Rane S.G. (2006). Nucleocytoplasmic shuttling of the retinoblastoma tumor suppressor protein via Cdk phosphorylation-dependent nuclear export. J. Biol. Chem..

[26] Drucker E., Holzer K., Pusch S., Winkler J., Calvisi D.F., Eiteneuer E., Herpel E., Goeppert B., Roessler S., Ori A. (2019). Karyopherin alpha2-dependent import of E2F1 and TFDP1 maintains protumorigenic stathmin expression in liver cancer. Cell Commun. Signal..

[27] Engeland K. (2018). Cell cycle arrest through indirect transcriptional repression by p53: I have a DREAM. Cell Death Differ..

[28] Oren M. (2003). Decision making by p53: Life, death and cancer. Cell Death Differ..

[29] Lahalle A., Lacroix M., De Blasio C., Cisse M.Y., Linares L.K., Le Cam L. (2021). The p53 Pathway and Metabolism: The Tree That Hides the Forest. Cancers.

[30] Yu L., Wu M., Zhu G., Xu Y. (2021). Emerging Roles of the Tumor Suppressor p53 in Metabolism. Front. Cell Dev. Biol..

[31] Nachmias B., Schimmer A.D. (2020). Targeting nuclear import and export in hematological malignancies. Leukemia.

[32] Miles X., Vandevoorde C., Hunter A., Bolcaen J. (2021). MDM2/X Inhibitors as Radiosensitizers for Glioblastoma Targeted Therapy. Front. Oncol..

[33] Porta C., Paglino C., Mosca A. (2014). Targeting PI3K/Akt/mTOR Signaling in Cancer. Front. Oncol..

[34] Vo V.A., Lee J.W., Lee H.J., Chun W., Lim S.Y., Kim S.S. (2014). Inhibition of JNK potentiates temozolomide-induced cytotoxicity in U87MG glioblastoma cells via suppression of Akt phosphorylation. Anticancer Res..

[35] Gravina G.L., Senapedis W., McCauley D., Baloglu E., Shacham S., Festuccia C. (2014). Nucleo-cytoplasmic transport as a therapeutic target of cancer. J. Hematol. Oncol..

[36] Sun Y., Li W., Li X., Zheng H., Qiu Y., Yang H. (2021). Oncogenic role of karyopherin alpha2 (KPNA2) in human tumors: A pan-cancer analysis. Comput. Biol. Med..

[37] Martinez-Olivera R., Datsi A., Stallkamp M., Koller M., Kohtz I., Pintea B., Gousias K. (2018). Silencing of the nucleocytoplasmic shuttling protein karyopherin a2 promotes cell-cycle arrest and apoptosis in glioblastoma multiforme. Oncotarget.

[38] Gousias K., Niehusmann P., Gielen G.H., Simon M. (2014). Karyopherin a2 and chromosome region maintenance protein 1 expression in meningiomas: Novel biomarkers for recurrence and malignant progression. J. Neurooncol..

[39] Gousias K., Becker A.J., Simon M., Niehusmann P. (2012). Nuclear karyopherin a2: A novel biomarker for infiltrative astrocytomas. J. Neurooncol..

[40] Hill J.R., Kuriyama N., Kuriyama H., Israel M.A. (1999). Molecular genetics of brain tumors. Arch. Neurol..

[41] Zhang Y., Dube C., Gibert M., Cruickshanks N., Wang B., Coughlan M., Yang Y., Setiady I., Deveau C., Saoud K. (2018). The p53 Pathway in Glioblastoma. Cancers.

[42] Xiong Y., Zhang Y., Xiong S., Williams-Villalobo A.E. (2020). A Glance of p53 Functions in Brain Development, Neural Stem Cells, and Brain Cancer. Biology.

[43] Uno M., Oba-Shinjo S.M., de Aguiar P.H., Leite C.C., Rosemberg S., Miura F.K., Junior R.M., Scaff M., Nagahashi Marie S.K. (2005). Detection of somatic TP53 splice site mutations in diffuse astrocytomas. Cancer Lett..

[44] Marei H.E., Althani A., Afifi N., Hasan A., Caceci T., Pozzoli G., Morrione A., Giordano A., Cenciarelli C. (2021). p53 signaling in cancer progression and therapy. Cancer Cell Int..

[45] Duffy M.J., Synnott N.C., O’Grady S., Crown J. (2020). Targeting p53 for the treatment of cancer. Semin. Cancer Biol..

[46] Jesionek-Kupnicka D., Szybka M., Malachowska B., Fendler W., Potemski P., Piaskowski S., Jaskolski D., Papierz W., Skowronski W., Och W. (2014). TP53 promoter methylation in primary glioblastoma: Relationship with TP53 mRNA and protein expression and mutation status. DNA Cell Biol..

[47] Reifenberger G., Liu L., Ichimura K., Schmidt E.E., Collins V.P. (1993). Amplification and overexpression of the MDM2 gene in a subset of human malignant gliomas without p53 mutations. Cancer Res..

[48] Riemenschneider M.J., Buschges R., Wolter M., Reifenberger J., Bostrom J., Kraus J.A., Schlegel U., Reifenberger G. (1999). Amplification and overexpression of the MDM4 (MDMX) gene from 1q32 in a subset of malignant gliomas without TP53 mutation or MDM2 amplification. Cancer Res..

[49] Nakamura M., Watanabe T., Klangby U., Asker C., Wiman K., Yonekawa Y., Kleihues P., Ohgaki H. (2001). p14ARF deletion and methylation in genetic pathways to glioblastomas. Brain Pathol..

[50] Squatrito M., Brennan C.W., Helmy K., Huse J.T., Petrini J.H., Holland E.C. (2010). Loss of ATM/Chk2/p53 pathway components accelerates tumor development and contributes to radiation resistance in gliomas. Cancer Cell.

[51] Viotti J., Duplan E., Caillava C., Condat J., Goiran T., Giordano C., Marie Y., Idbaih A., Delattre J.Y., Honnorat J. (2014). Glioma tumor grade correlates with parkin depletion in mutant p53-linked tumors and results from loss of function of p53 transcriptional activity. Oncogene.

[52] Lee J.S., Xiao J., Patel P., Schade J., Wang J., Deneen B., Erdreich-Epstein A., Song H.R. (2014). A novel tumor-promoting role for nuclear factor IA in glioblastomas is mediated through negative regulation of p53, p21, and PAI1. Neuro-Oncology.

[53] Zhou X., Wu W., Zeng A., Nie E., Jin X., Yu T., Zhi T., Jiang K., Wang Y., Zhang J. (2017). MicroRNA-141-3p promotes glioma cell growth and temozolomide resistance by directly targeting p53. Oncotarget.

[54] Stegh A.H., Brennan C., Mahoney J.A., Forloney K.L., Jenq H.T., Luciano J.P., Protopopov A., Chin L., Depinho R.A. (2010). Glioma oncoprotein Bcl2L12 inhibits the p53 tumor suppressor. Genes Dev..

[55] Stegh A.H., Kim H., Bachoo R.M., Forloney K.L., Zhang J., Schulze H., Park K., Hannon G.J., Yuan J., Louis D.N. (2007). Bcl2L12 inhibits post-mitochondrial apoptosis signaling in glioblastoma. Genes Dev..

[56] Fukaya R., Ohta S., Yaguchi T., Matsuzaki Y., Sugihara E., Okano H., Saya H., Kawakami Y., Kawase T., Yoshida K. (2016). MIF Maintains the Tumorigenic Capacity of Brain Tumor-Initiating Cells by Directly Inhibiting p53. Cancer Res..

[57] Cancer Genome Atlas Research N. (2008). Comprehensive genomic characterization defines human glioblastoma genes and core pathways. Nature.

[58] Grzmil M., Hemmings B.A. (2010). Deregulated signalling networks in human brain tumours. Biochim. Biophys. Acta.

[59] Zanotto-Filho A., Braganhol E., Edelweiss M.I., Behr G.A., Zanin R., Schroder R., Simoes-Pires A., Battastini A.M., Moreira J.C. (2012). The curry spice curcumin selectively inhibits cancer cells growth in vitro and in preclinical model of glioblastoma. J. Nutr. Biochem..

[60] Koul D. (2008). PTEN signaling pathways in glioblastoma. Cancer Biol. Ther..

[61] Yang K., Liu Y., Liu Z., Liu J., Liu X., Chen X., Li C., Zeng Y. (2013). p38gamma overexpression in gliomas and its role in proliferation and apoptosis. Sci. Rep..

[62] Matsuda K., Sato A., Okada M., Shibuya K., Seino S., Suzuki K., Watanabe E., Narita Y., Shibui S., Kayama T. (2012). Targeting JNK for therapeutic depletion of stem-like glioblastoma cells. Sci. Rep..

[63] Raychaudhuri B., Han Y., Lu T., Vogelbaum M.A. (2007). Aberrant constitutive activation of nuclear factor kappaB in glioblastoma multiforme drives invasive phenotype. J. Neurooncol..

[64] Smith D., Shimamura T., Barbera S., Bejcek B.E. (2008). NF-kappaB controls growth of glioblastomas/astrocytomas. Mol. Cell. Biochem..

[65] Cahill K.E., Morshed R.A., Yamini B. (2016). Nuclear factor-kappaB in glioblastoma: Insights into regulators and targeted therapy. Neuro-Oncology.

[66] Killela P.J., Reitman Z.J., Jiao Y., Bettegowda C., Agrawal N., Diaz L.A., Friedman A.H., Friedman H., Gallia G.L., Giovanella B.C. (2013). TERT promoter mutations occur frequently in gliomas and a subset of tumors derived from cells with low rates of self-renewal. Proc. Natl. Acad. Sci. USA.

[67] Brennan C.W., Verhaak R.G., McKenna A., Campos B., Noushmehr H., Salama S.R., Zheng S., Chakravarty D., Sanborn J.Z., Berman S.H. (2013). The somatic genomic landscape of glioblastoma. Cell.

[68] Simon M., Hosen I., Gousias K., Rachakonda S., Heidenreich B., Gessi M., Schramm J., Hemminki K., Waha A., Kumar R. (2015). TERT promoter mutations: A novel independent prognostic factor in primary glioblastomas. Neuro-Oncology.

[69] Stupp R., Taillibert S., Kanner A., Read W., Steinberg D., Lhermitte B., Toms S., Idbaih A., Ahluwalia M.S., Fink K. (2017). Effect of Tumor-Treating Fields Plus Maintenance Temozolomide vs. Maintenance Temozolomide Alone on Survival in Patients With Glioblastoma: A Randomized Clinical Trial. JAMA.

[70] Biau J., Chautard E., Berthault N., de Koning L., Court F., Pereira B., Verrelle P., Dutreix M. (2019). Combining the DNA Repair Inhibitor Dbait With Radiotherapy for the Treatment of High Grade Glioma: Efficacy and Protein Biomarkers of Resistance in Preclinical Models. Front. Oncol..

[71] Maier P., Hartmann L., Wenz F., Herskind C. (2016). Cellular Pathways in Response to Ionizing Radiation and Their Targetability for Tumor Radiosensitization. Int. J. Mol. Sci..

[72] Warters R.L., Hofer K.G., Harris C.R., Smith J.M. (1978). Radionuclide toxicity in cultured mammalian cells: Elucidation of the primary site of radiation damage. Curr. Top. Radiat. Res. Q..

[73] Franken N.A., ten Cate R., Krawczyk P.M., Stap J., Haveman J., Aten J., Barendsen G.W. (2011). Comparison of RBE values of high-LET alpha-particles for the induction of DNA-DSBs, chromosome aberrations and cell reproductive death. Radiat. Oncol..

[74] Zhu P., Zhu J.J. (2017). Tumor treating fields: A novel and effective therapy for glioblastoma: Mechanism, efficacy, safety and future perspectives. Chin. Clin. Oncol..

[75] Gera N., Yang A., Holtzman T.S., Lee S.X., Wong E.T., Swanson K.D. (2015). Tumor treating fields perturb the localization of septins and cause aberrant mitotic exit. PLoS ONE.

[76] Wu S., Li X., Gao F., de Groot J.F., Koul D., Yung W.K.A. (2021). PARP-mediated PARylation of MGMT is critical to promote repair of temozolomide-induced O6-methylguanine DNA damage in glioblastoma. Neuro-Oncology.

[77] Hegi M.E., Diserens A.C., Gorlia T., Hamou M.F., de Tribolet N., Weller M., Kros J.M., Hainfellner J.A., Mason W., Mariani L. (2005). MGMT gene silencing and benefit from temozolomide in glioblastoma. N. Engl. J. Med..

[78] Weller M., Le Rhun E. (2020). How did lomustine become standard of care in recurrent glioblastoma?. Cancer Treat. Rev..

[79] Herrlinger U., Tzaridis T., Mack F., Steinbach J.P., Schlegel U., Sabel M., Hau P., Kortmann R.D., Krex D., Grauer O. (2019). Lomustine-temozolomide combination therapy versus standard temozolomide therapy in patients with newly diagnosed glioblastoma with methylated MGMT promoter (CeTeG/NOA-09): A randomised, open-label, phase 3 trial. Lancet.

[80] Esteller M., Garcia-Foncillas J., Andion E., Goodman S.N., Hidalgo O.F., Vanaclocha V., Baylin S.B., Herman J.G. (2000). Inactivation of the DNA-repair gene MGMT and the clinical response of gliomas to alkylating agents. N. Engl. J. Med..

[81] Patnaik A., Rosen L.S., Tolaney S.M., Tolcher A.W., Goldman J.W., Gandhi L., Papadopoulos K.P., Beeram M., Rasco D.W., Hilton J.F. (2016). Efficacy and Safety of Abemaciclib, an Inhibitor of CDK4 and CDK6, for Patients with Breast Cancer, Non-Small Cell Lung Cancer, and Other Solid Tumors. Cancer Discov..

[82] Tong H., Zhao K., Zhang J., Zhu J., Xiao J. (2019). YB-1 modulates the drug resistance of glioma cells by activation of MDM2/p53 pathway. Drug Des. Devel. Ther..

[83] Wu Q., Cao Z., Xiao W., Zhu L., Xie Q., Li L., Zhang B., Zhao W. (2018). Study on Therapeutic Action and Mechanism of TMZ Combined with RITA Against Glioblastoma. Cell. Physiol. Biochem..

[84] Rouland L., Duplan E., Ramos Dos Santos L., Bernardin A., Katula K.S., Manfioletti G., Idbaih A., Checler F., Alves da Costa C. (2021). Therapeutic potential of parkin as a tumor suppressor via transcriptional control of cyclins in glioblastoma cell and animal models. Theranostics.

[85] Clark P.A., Bhattacharya S., Elmayan A., Darjatmoko S.R., Thuro B.A., Yan M.B., van Ginkel P.R., Polans A.S., Kuo J.S. (2017). Resveratrol targeting of AKT and p53 in glioblastoma and glioblastoma stem-like cells to suppress growth and infiltration. J. Neurosurg..

[86] Goel S., DeCristo M.J., McAllister S.S., Zhao J.J. (2018). CDK4/6 Inhibition in Cancer: Beyond Cell Cycle Arrest. Trends Cell Biol..

[87] Le Rhun E., Preusser M., Roth P., Reardon D.A., van den Bent M., Wen P., Reifenberger G., Weller M. (2019). Molecular targeted therapy of glioblastoma. Cancer Treat Rev.

[88] Su C.C., Wang M.J., Chiu T.L. (2010). The anti-cancer efficacy of curcumin scrutinized through core signaling pathways in glioblastoma. Int. J. Mol. Med..

[89] Yeh L.T., Hsu L.S., Chung Y.H., Chen C.J. (2020). Tectorigenin Inhibits Glioblastoma Proliferation by G0/G1 Cell Cycle Arrest. Medicina.

[90] Karmakar S., Banik N.L., Ray S.K. (2007). Curcumin suppressed anti-apoptotic signals and activated cysteine proteases for apoptosis in human malignant glioblastoma U87MG cells. Neurochem. Res..

[91] Doan P., Musa A., Candeias N.R., Emmert-Streib F., Yli-Harja O., Kandhavelu M. (2019). Alkylaminophenol Induces G1/S Phase Cell Cycle Arrest in Glioblastoma Cells Through p53 and Cyclin-Dependent Kinase Signaling Pathway. Front. Pharmacol..

[92] Alexiou G.A., Lazari D., Markopoulos G., Vartholomatos E., Hodaj E., Galani V., Kyritsis A.P. (2017). Moschamine inhibits proliferation of glioblastoma cells via cell cycle arrest and apoptosis. Tumour Biol..

[93] Wadhwa E., Nicolaides T. (2016). Bromodomain Inhibitor Review: Bromodomain and Extra-terminal Family Protein Inhibitors as a Potential New Therapy in Central Nervous System Tumors. Cureus.

[94] Cheung K.L., Kim C., Zhou M.M. (2021). The Functions of BET Proteins in Gene Transcription of Biology and Diseases. Front. Mol. Biosci..

[95] Wen N., Guo B., Zheng H., Xu L., Liang H., Wang Q., Wang D., Chen X., Zhang S., Li Y. (2019). Bromodomain inhibitor jq1 induces cell cycle arrest and apoptosis of glioma stem cells through the VEGF/PI3K/AKT signaling pathway. Int. J. Oncol..

[96] Gareev I., Beylerli O., Liang Y., Xiang H., Liu C., Xu X., Yuan C., Ahmad A., Yang G. (2021). The Role of MicroRNAs in Therapeutic Resistance of Malignant Primary Brain Tumors. Front. Cell Dev. Biol..

[97] Gallia G.L., Holdhoff M., Brem H., Joshi A.D., Hann C.L., Bai R.Y., Staedtke V., Blakeley J.O., Sengupta S., Jarrell T.C. (2021). Mebendazole and temozolomide in patients with newly diagnosed high-grade gliomas: Results of a phase 1 clinical trial. Neurooncol. Adv..

[98] Hu Y., Zhou W., Xue Z., Liu X., Feng Z., Zhang Y., Zhang X., Liu X., Li W., Zhang Q. (2022). Thiabendazole inhibits glioblastoma cell proliferation and invasion targeting MCM2. J. Pharmacol. Exp. Ther..

[99] Zhou X., Liu J., Zhang J., Wei Y., Li H. (2018). Flubendazole inhibits glioma proliferation by G2/M cell cycle arrest and pro-apoptosis. Cell Death Discov..

[100] Stummer W., Pichlmeier U., Meinel T., Wiestler O.D., Zanella F., Reulen H.J., Group A.L.-G.S. (2006). Fluorescence-guided surgery with 5-aminolevulinic acid for resection of malignant glioma: A randomised controlled multicentre phase III trial. Lancet Oncol..

[101] Schipmann S., Muther M., Stogbauer L., Zimmer S., Brokinkel B., Holling M., Grauer O., Suero Molina E., Warneke N., Stummer W. (2020). Combination of ALA-induced fluorescence-guided resection and intraoperative open photodynamic therapy for recurrent glioblastoma: Case series on a promising dual strategy for local tumor control. J. Neurosurg..

[102] Hemmerlein B., Weseloh R.M., Mello de Queiroz F., Knotgen H., Sanchez A., Rubio M.E., Martin S., Schliephacke T., Jenke M., Heinz Joachim R. (2006). Overexpression of Eag1 potassium channels in clinical tumours. Mol. Cancer.

[103] Pollak J., Rai K.G., Funk C.C., Arora S., Lee E., Zhu J., Price N.D., Paddison P.J., Ramirez J.M., Rostomily R.C. (2017). Ion channel expression patterns in glioblastoma stem cells with functional and therapeutic implications for malignancy. PLoS ONE.

[104] Weaver A.K., Liu X., Sontheimer H. (2004). Role for calcium-activated potassium channels (BK) in growth control of human malignant glioma cells. J. Neurosci. Res..

[105] Green A.L., Ramkissoon S.H., McCauley D., Jones K., Perry J.A., Hsu J.H., Ramkissoon L.A., Maire C.L., Hubbell-Engler B., Knoff D.S. (2015). Preclinical antitumor efficacy of selective exportin 1 inhibitors in glioblastoma. Neuro-Oncology.

[106] Cruz Da Silva E., Mercier M.C., Etienne-Selloum N., Dontenwill M., Choulier L. (2021). A Systematic Review of Glioblastoma-Targeted Therapies in Phases II, III, IV Clinical Trials. Cancers.

[107] Costa B., Bendinelli S., Gabelloni P., Da Pozzo E., Daniele S., Scatena F., Vanacore R., Campiglia P., Bertamino A., Gomez-Monterrey I. (2013). Human glioblastoma multiforme: p53 reactivation by a novel MDM2 inhibitor. PLoS ONE.

[108] Verreault M., Schmitt C., Goldwirt L., Pelton K., Haidar S., Levasseur C., Guehennec J., Knoff D., Labussiere M., Marie Y. (2016). Preclinical Efficacy of the MDM2 Inhibitor RG7112 in MDM2-Amplified and TP53 Wild-type Glioblastomas. Clin. Cancer Res..

[109] Tien A.C., Li J., Bao X., Derogatis A., Kim S., Mehta S., Sanai N. (2019). A Phase 0 Trial of Ribociclib in Recurrent Glioblastoma Patients Incorporating a Tumor Pharmacodynamic- and Pharmacokinetic-Guided Expansion Cohort. Clin. Cancer Res..

[110] Nguyen L.V., Searle K., Jerzak K.J. (2019). Central nervous system-specific efficacy of CDK4/6 inhibitors in randomized controlled trials for metastatic breast cancer. Oncotarget.

[111] Michaud K., Solomon D.A., Oermann E., Kim J.S., Zhong W.Z., Prados M.D., Ozawa T., James C.D., Waldman T. (2010). Pharmacologic inhibition of cyclin-dependent kinases 4 and 6 arrests the growth of glioblastoma multiforme intracranial xenografts. Cancer Res..

[112] Jermakowicz A.M., Rybin M.J., Suter R.K., Sarkaria J.N., Zeier Z., Feng Y., Ayad N.G. (2021). The novel BET inhibitor UM-002 reduces glioblastoma cell proliferation and invasion. Sci. Rep..

[113] Dey A., Nishiyama A., Karpova T., McNally J., Ozato K. (2009). Brd4 marks select genes on mitotic chromatin and directs postmitotic transcription. Mol. Biol. Cell.

[114] Wang R., Gurguis C.I., Gu W., Ko E.A., Lim I., Bang H., Zhou T., Ko J.H. (2015). Ion channel gene expression predicts survival in glioma patients. Sci. Rep..

[115] Bai R.Y., Staedtke V., Aprhys C.M., Gallia G.L., Riggins G.J. (2011). Antiparasitic mebendazole shows survival benefit in 2 preclinical models of glioblastoma multiforme. Neuro-Oncology.

[116] Ren L.W., Li W., Zheng X.J., Liu J.Y., Yang Y.H., Li S., Zhang S., Fu W.Q., Xiao B., Wang J.H. (2022). Benzimidazoles induce concurrent apoptosis and pyroptosis of human glioblastoma cells via arresting cell cycle. Acta Pharmacol. Sin..

[117] Weissenrieder J.S., Reed J.L., Green M.V., Moldovan G.L., Koubek E.J., Neighbors J.D., Hohl R.J. (2020). The Dopamine D2 Receptor Contributes to the Spheroid Formation Behavior of U87 Glioblastoma Cells. Pharmacology.

[118] Lin W.Z., Liu Y.C., Lee M.C., Tang C.T., Wu G.J., Chang Y.T., Chu C.M., Shiau C.Y. (2022). From GWAS to drug screening: Repurposing antipsychotics for glioblastoma. J. Transl. Med..

[119] Papadopoulos F., Isihou R., Alexiou G.A., Tsalios T., Vartholomatos E., Markopoulos G.S., Sioka C., Tsekeris P., Kyritsis A.P., Galani V. (2020). Haloperidol Induced Cell Cycle Arrest and Apoptosis in Glioblastoma Cells. Biomedicines.

[120] Jalili-Nik M., Abbasinezhad-Moud F., Sahab-Negah S., Maghrouni A., Etezad Razavi M., Khaleghi Ghadiri M., Stummer W., Gorji A. (2021). Antitumor Effects of 5-Aminolevulinic Acid on Human Malignant Glioblastoma Cells. Int. J. Mol. Sci..

[121] Griffin M., Khan R., Basu S., Smith S. (2020). Ion Channels as Therapeutic Targets in High Grade Gliomas. Cancers.

[122] Alptekin M., Eroglu S., Tutar E., Sencan S., Geyik M.A., Ulasli M., Demiryurek A.T., Camci C. (2015). Gene expressions of TRP channels in glioblastoma multiforme and relation with survival. Tumour Biol..

[123] Staudacher I., Jehle J., Staudacher K., Pledl H.W., Lemke D., Schweizer P.A., Becker R., Katus H.A., Thomas D. (2014). HERG K+ channel-dependent apoptosis and cell cycle arrest in human glioblastoma cells. PLoS ONE.

[124] Lassman A.B., Wen P.Y., van den Bent M.J., Plotkin S.R., Walenkamp A.M.E., Green A.L., Li K., Walker C.J., Chang H., Tamir S. (2021). A Phase 2 Study of the Efficacy and Safety of Oral Selinexor in Recurrent Glioblastoma. Clin. Cancer Res..

